# A drug target slim: using gene ontology and gene ontology annotations to navigate protein-ligand target space in ChEMBL

**DOI:** 10.1186/s13326-016-0102-0

**Published:** 2016-09-27

**Authors:** Prudence Mutowo, A. Patrícia Bento, Nathan Dedman, Anna Gaulton, Anne Hersey, Jane Lomax, John P. Overington

**Affiliations:** 1 European Molecular Biology Laboratory, European Bioinformatics Institute (EMBL-EBI), Wellcome Trust Genome Campus, Hinxton, Cambridge, CB10 1SD UK; 2 Wellcome Trust Sanger Institute, Wellcome Trust Genome Campus, Hinxton, Cambridge, CB10 1SA UK

**Keywords:** Ontologies, Bioinformatics, Drug discovery, Database, Biology, Protein

## Abstract

**Background:**

The process of discovering new drugs is a lengthy, time-consuming and expensive process. Modern day drug discovery relies heavily on the rapid identification of novel ‘targets’, usually proteins that can be modulated by small molecule drugs to cure or minimise the effects of a disease. Of the 20,000 proteins currently reported as comprising the human proteome, just under a quarter of these can potentially be modulated by known small molecules Storing information in curated, actively maintained drug discovery databases can help researchers access current drug discovery information quickly. However with the increase in the amount of data generated from both experimental and *in silico* efforts, databases can become very large very quickly and information retrieval from them can become a challenge. The development of database tools that facilitate rapid information retrieval is important to keep up with the growth of databases.

**Description:**

We have developed a Gene Ontology-based navigation tool (Gene Ontology Tree) to help users retrieve biological information to single protein targets in the ChEMBL drug discovery database. 99 % of single protein targets in ChEMBL have at least one GO annotation associated with them. There are 12,500 GO terms associated to 6200 protein targets in the ChEMBL database resulting in a total of 140,000 annotations. The slim we have created, the ‘ChEMBL protein target slim’ allows broad categorisation of the biology of 90 % of the protein targets using just 300 high level, informative GO terms.

We used the GO slim method of assigning fewer higher level GO groupings to numerous very specific lower level terms derived from the GOA to describe a set of GO terms relevant to proteins in ChEMBL. We then used the slim created to provide a web based tool that allows a quick and easy navigation of protein target space. Terms from the GO are used to capture information on protein molecular function, biological process and subcellular localisations. The ChEMBL database also provides compound information for small molecules that have been tested for their effects on these protein targets. The ‘ChEMBL protein target slim’ provides a means of firstly describing the biology of protein drug targets and secondly allows users to easily establish a connection between biological and chemical information regarding drugs and drug targets in ChEMBL.

The ‘ChEMBL protein target slim’ is available as a browsable ‘Gene Ontology Tree’ on the ChEMBL site under the browse targets tab (https://www.ebi.ac.uk/chembl/target/browser). A ChEMBL protein target slim OBO file containing the GO slim terms pertinent to ChEMBL is available from the GOC website (http://geneontology.org/page/go-slim-and-subset-guide).

**Conclusions:**

We have created a protein target navigation tool based on the ‘ChEMBL protein target slim’. The ‘ChEMBL protein target slim’ provides a way of browsing protein targets in ChEMBL using high level GO terms that describe the molecular functions, processes and subcellular localisations of protein drug targets in drug discovery. The tool also allows user to establish a link between ontological groupings representing protein target biology to relevant compound information in ChEMBL. We have demonstrated by the use of a simple example how the ‘ChEMBL protein target slim’ can be used to link biological processes with drug information based on the information in the ChEMBL database. The tool has potential to aid in areas of drug discovery such as drug repurposing studies or drug-disease-protein pathways.

## Background

The use of small molecules in alleviating symptoms in a disease state is generally evaluated against a protein target [1]. The human proteome is reported to have around 20,000 proteins [2] with literature sources reporting a great variation in the number of proteins deemed to be druggable [3, 4]. The biology of the druggable proteome (alternatively the druggable genome) is usually described in terms of distinct and well-studied protein families. Protein family classification can be used together with protein-centric bio-ontologies to better understand the characteristics of a drug target of interest.

ChEMBL is a manually curated, freely available resource containing bioactive ligands with drug-like properties as well as quantitative bioassay results and the biological targets of these molecules [5]. Biological targets reported in ChEMBL assays include nucleic acids, cell-lines, tissues, subcellular-fractions, whole organisms and proteins. Protein targets are the largest portion of targets in ChEMBL. This target group is further divided into single protein targets, protein complexes, protein families, and protein-protein interactions. Compound information to ChEMBL protein targets is obtained by manual curation of selected published medicinal chemistry literature and data depositions from data sharing partnerships.

There has been a steady increase of data in ChEMBL over time. There has been a fourfold increase in the total numbers of assays from the first ChEMBL release (ChEMBL1) to the current release (ChEMBL21) which has 1.2 million assays. The number of single protein targets doubled with the first release having 3222 single protein targets to just over 6000 in the current release. Standardising protein information in ChEMBL is useful to facilitate ease of protein target retrieval. Single protein targets in the database are cross-referenced to a variety of protein property descriptors including Gene Ontology (GO) based annotations. GO is a set of concepts, structured as a graph or tree, that provide a controlled and concise way of capturing the processes, molecular functions and subcellular localisations of gene products in this case proteins [6]. An annotation is an evidence-based assertion created to capture biological information about a protein [7]. GO annotations to protein targets in ChEMBL are obtained from the GO consortium database [8]. These annotations provide useful insight into the biology of proteins in drug discovery. GO annotations vary in their information content depending on the specificity of the term used in annotation. Some annotations contain very specific and fine-grained information about a protein while others contain broad, high level information. Comparing, grouping or searching through protein targets annotated at different levels of GO information content can be time consuming and challenging. GO slims are often used to allow comparison of protein information captured at different levels of the GO.

A GO slim is a high-level subset of the GO created by collapsing specific terms and ‘mapping’ them to their higher level parent terms using the parent–child hierarchies inherent in the GO. GO slimming allows for a representation of biological information by using high level terms that provide a broad overview of the biology [8]. GO slims are typically generated for specific organism or particular areas of scientific interest and have been used to aid visualisation, exploration and summarization of GO functional data [9, 10]. We have created a ‘ChEMBL protein target slim’ to allow users to easily access the biological information to targets with GO annotation.

## Construction and content

### Creating the ‘ChEMBL protein target slim’

We created the ChEMBL target slim by retrieving all relevant annotations to single protein targets in ChEMBL using the QuickGO tool [11]. QuickGO is a web browser for GO terms and annotations. GO terms in QuickGO are identified by an alpha numeric identifier, a term definition and relationships established between a specific term and other terms in GO. Annotations retrieved from QuickGO are obtained from the Gene Ontology consortium GO database and are created by consortium members. We retrieved all annotations to the protein set across all evidence codes. The output was downloaded as a Gene Association File (GAF) which contains protein accessions and GO term information.

We used the generic GO term mapper tool [12] to identify an initial high level set of GO terms representing the annotation information for our protein target set. The GO term mapper tool uses the map2slim algorithm in ‘count mode’ to identify high level term parent terms to terms in the annotation set. The slim terms suggested by the algorithm are grouped according to the number of proteins whose initial annotation has been mapped to a higher level GO term. GO terms with a high number of proteins mapped are incorporated in the slim while terms with no proteins are removed. We selected the Generic GO slim (version 1.2) as the reference slim for the selection of slim terms. This slim is not species specific.

From the output the term mapping we manually inspected and customised the slim to the ChEMBL protein target set as follows:

#### GO term refinement

We identified fine –grained annotations that could be mapped to higher level terms. One of the considerations made in this exercise was the information content of the higher level terms. An example being proteins annotated to granular protein binding terms like GO:0017124 SH3 domain binding were not mapped up to the higher level parent GO:0005515 protein binding due to loss of information content.

#### GO term selection

We assessed the number of accessions not mapped to any term in the initial reference generic GO slim with a view to customising the slim terms to reflect the biology of the protein annotations in the set. We manually added terms to the GO slim to address this.

We removed terms from the generic GO slim that did not have any annotations in the ChEMBL protein set.

This addition, removal and term refinement was done in several rounds of term-to-accession-mapped inspection until we obtained a set of slim terms providing a good coverage (in this case 90 %) of the protein targets in ChEMBL.

### Results

The resultant ChEMBL protein target slim generated contains a total of 300 high level GO terms representing the biology of 5600 protein targets in ChEMBL in the three areas of GO. In total, proteins from 532 different species are mapped to terms in the GO slim. The top ten species (in terms of number of proteins) in the current ChEMBL release are shown in Table 1.

**Table 1:** Proteins mapped to GO slim terms per species

Species	Proteins targets mapped to slim
Homo sapiens	3254
Rattus norvegicus	899
Mus musculus	828
Bos taurus	194
Sus scrofa	98
Escherichia coli K-12	74
Oryctolagus cuniculus	74
Mycobacterium tuberculosis	73
Saccharomyces cerevisiae S288c	70
Staphylococcus aureus	50

## Utility

Based on these slim term categories, we created a GO-based navigation tool which is available on the ChEMBL website. This tool termed the ‘Gene Ontology tree’ can be found by clicking on the ‘Browse Targets’ tab on the ChEMBL home page and selecting the radio button next to the tree name.

**Fig. 1:**
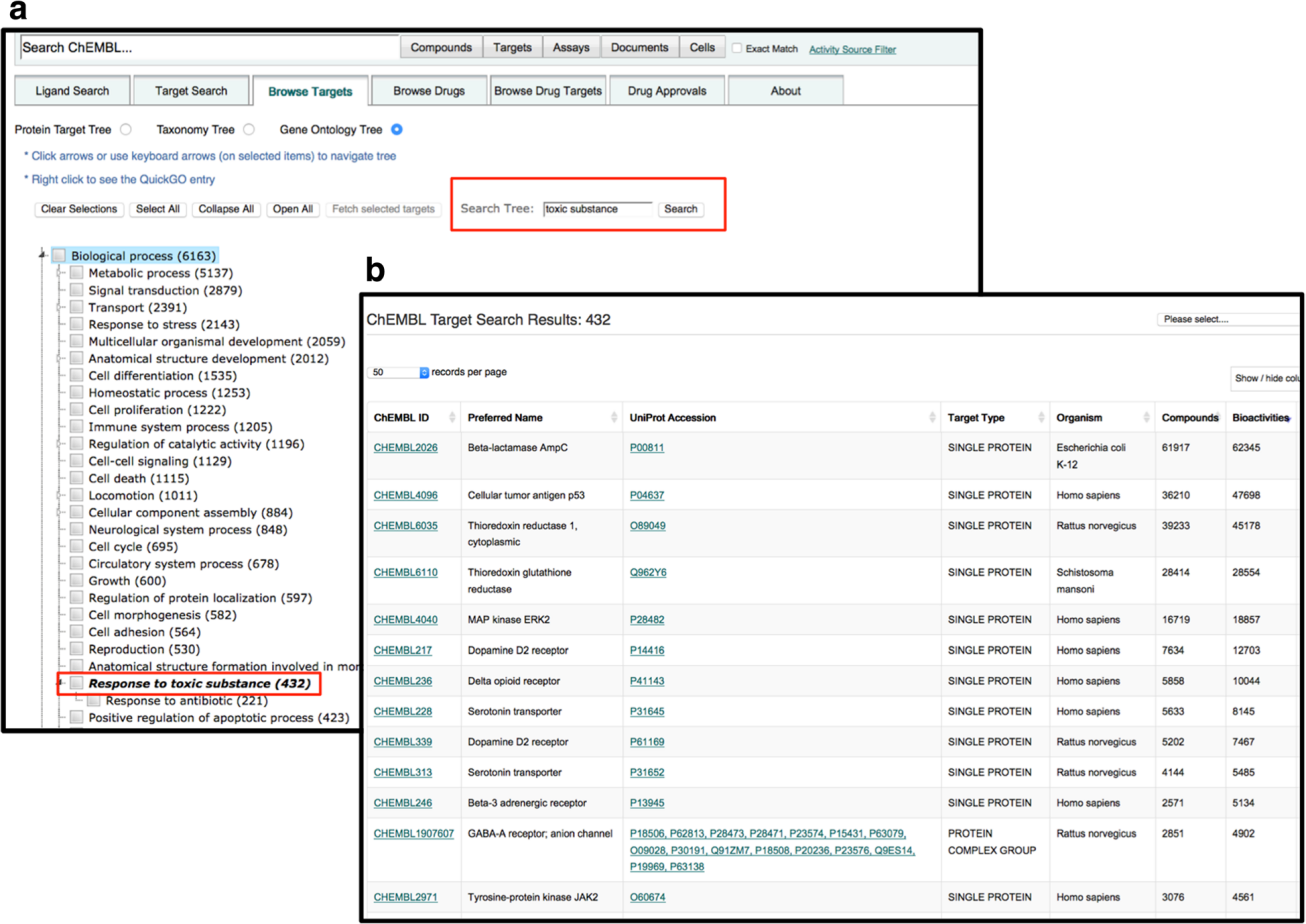
Searching the ChEMBL database using the GO tree to retrieve all proteins involved in response to toxic substance and their related compound and bioactivity information. Panel **a** shows the biological process node of the GO tree with a 'toxic substance' keyword search. Panel **b** shows the search output of the list of proteins annotated with the 'toxic substance' GO term

The two key functionalities of the GO tree are:

- *Protein target browsing by GO categories*
  The ChEMBL protein target slim in the form of a navigational GO tree also allows users to establish which processes or functions proteins are involved in by selecting the process and function nodes. The cellular component node provides a quick overview of the subcellular locations of protein targets as well as a link out to small molecules interaction with targets in a selected localisation (Fig. 1, Panel 1a). The numbers affiliated with each GO category on the tree allow a rapid assessment of which areas of biology have high proteins giving an indication of target prioritisation in drug discovery endeavours.
- *Searching for proteins and related compound information*
  The tree has a search functionality that allows users to search the database for protein information using a specific biological key word or phrase to retrieve all proteins targets annotated to that term as well as the compounds and bioactivities to the selected subset. Figure 1 shows how to use the tree to search for all proteins involved in response to toxic substance. By using a key phrase ‘toxic substance’ in the search box, the GO slim allows retrieval of all proteins annotated to GO:0009636 ‘response to toxic substance’ (Fig. 1, Panel 1a). The tree shows 432 protein targets annotated to this term as well as showing the more specific child terms of GO:0009636 which are ‘*GO:0046677 response to antibiotic*’. Right clicking on the GO term grouping information leads the user to a page containing all the protein targets annotated to that term as well as the compounds tested against them and the bioactivities reported for the assays (Fig. 1, panel 1b). A link exits to the QuickGO webpage to view the definition of the GO term of interest.

## Case study- using Gene Ontology and drug ATC information to further establish links between biological and chemical space in ChEMBL

Another useful application of the biological groupings created by the GO slim is the ability to provide insight into the biology of a group of proteins that are targets of drugs used for specific indications. The ChEMBL data base contains information to small molecules important in drug discovery. Small molecule to protein information links are primarily established by considering the bioassay that the two entities are reported together in. In addition, for FDA approved drugs, targets responsible for their efficacy (mechanism of action) are assigned manually. These high level drug classification categories were combined with the higher level GO classification categories for their targets to show the mechanism of action information displayed in Fig. 3.

The database also uses World Health Organisation anatomical therapeutic (ATC code) classifiers that describe the mechanism of action of a drug to group drugs in higher level categories. For examples drugs used against parasitic infections are grouped as anti-protozoals with an WHO ATC code of P01.

Figure 3 is a simple example of selecting a drug classification of interest from the ATC classification. We retrieved all drugs from ChEMBL that are used as Antineoplastic and Immunomodulating agents (WHO ATC classification L [13]), and the curated mechanism of action information and readily retrieving the biology of the targets of these drugs by using the GO slim categories that describe their assigned efficacy targets.

**Fig. 2:**
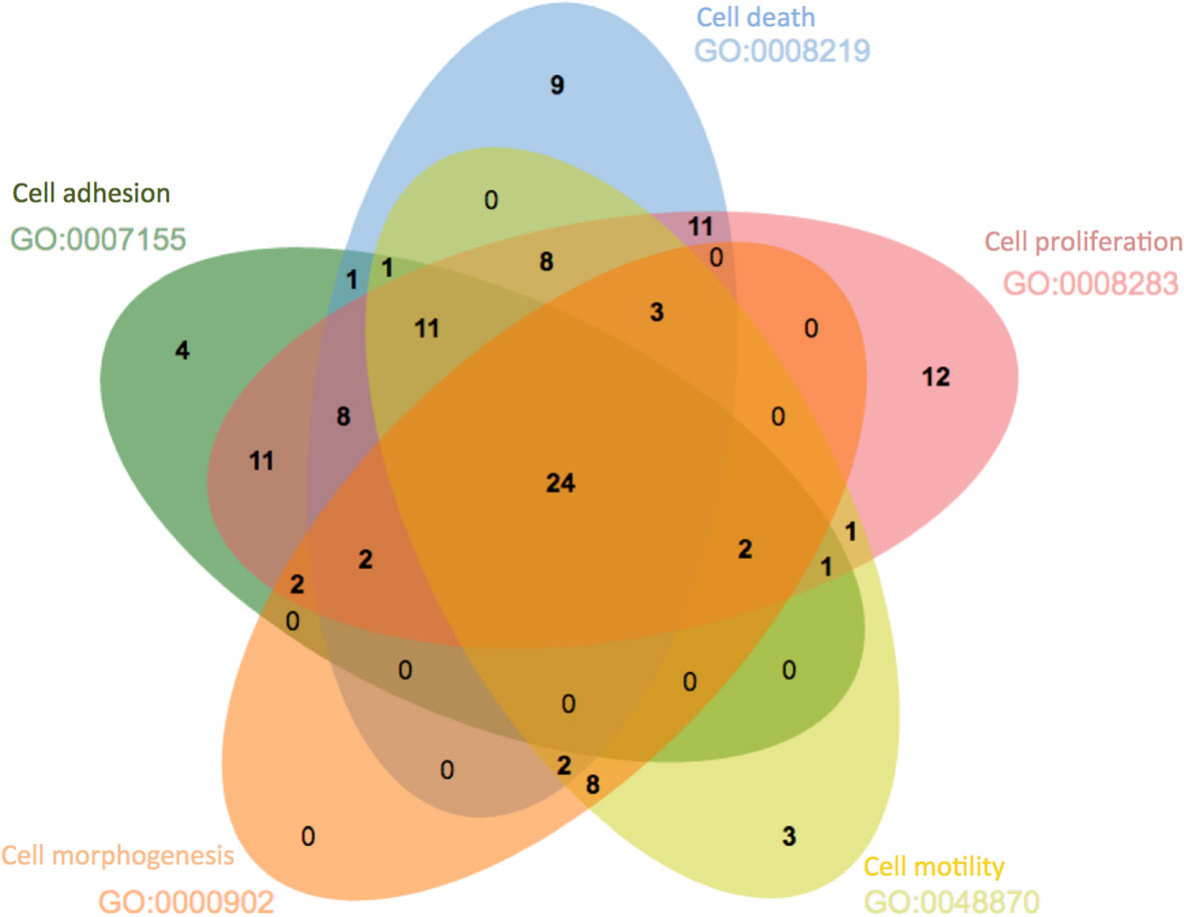
Number of drugs used as Antineoplastic and Immunomodulating Agents (ATC Class L) targeting proteins in 5 biological process categories generated using the ChEMBL slim

**Fig. 3:**
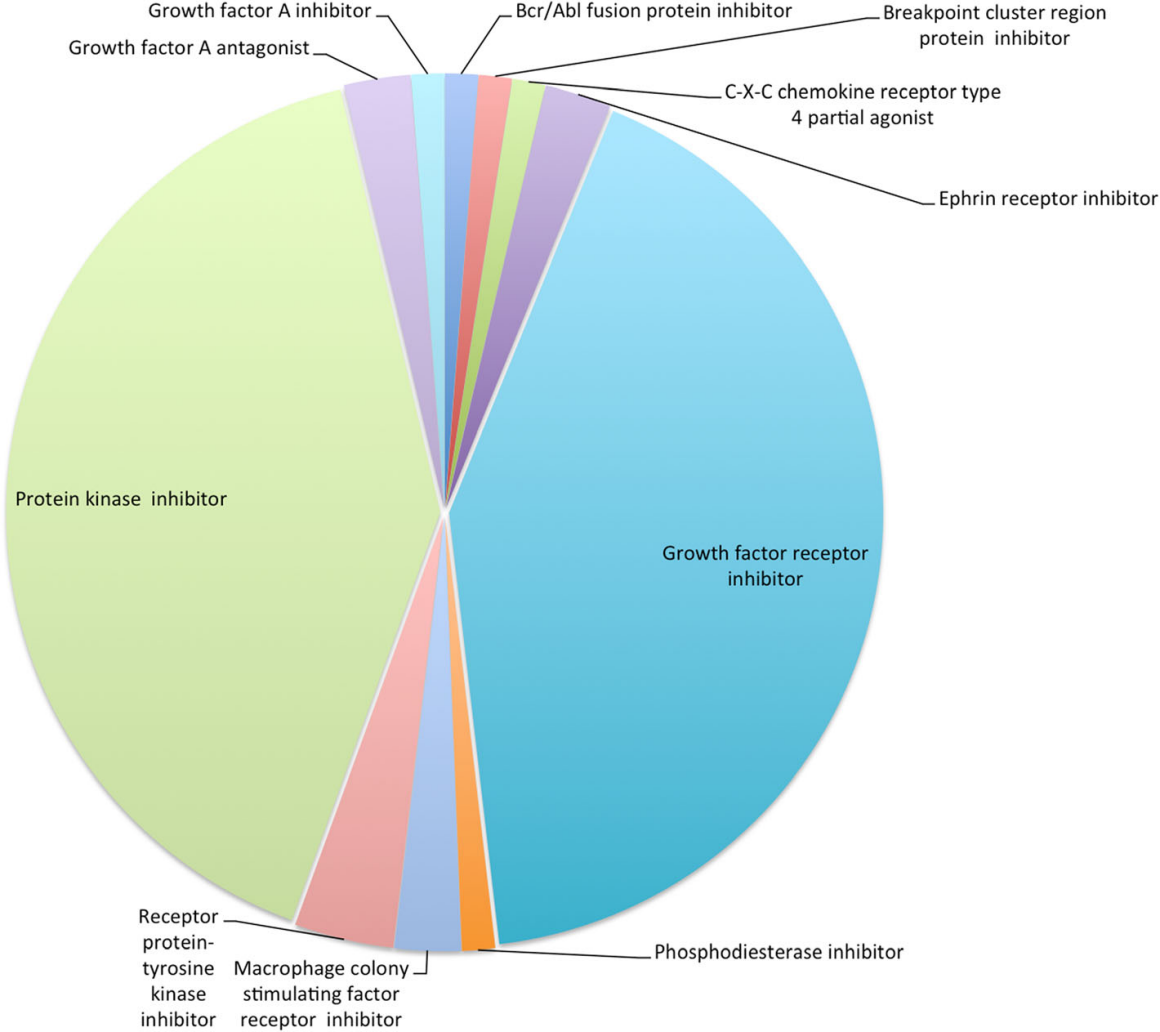
Mechanism of action for drugs at intersection of protein GO categories

The targets of all therapeutic drugs in release 20 of ChEMBL consists of 1179 individual proteins. Of this number, 196 proteins are targets of drugs in the ATC L class [13]. We used the ChEMBL slim to navigate the biological processes that these proteins are involved in. Figure 2 shows a venn diagram [14] of the 5 GO biological process categories for these proteins as cell death, cell motility, cell morphogenesis, cell proliferation and cell death. The number of drugs that modulate the proteins in each of the groupings are shown in the venn diagram sets. It is immediately apparent that 24 drugs have targets represented in all 5 categories of biological grouping. Using the drug mechanism of action information in ChEMBL we probed this set of 24 drugs on mechanism and the two main mechanism of actions shown are protein kinase inhibitor action and growth factor receptor inhibition (Fig. 3).

Considering higher level drug and target classification can give broad insight into the biology for protein targets of drugs in the same classification.

## Discussion

The existence of large numbers of protein annotations in the GO database provides a useful resource for computational querying of protein sets annotated in this way. The fact that not all GO annotations are made to the same level of term specificity can make it problematic to query protein sets annotated with this way. GO slims are a useful of compressing annotation information to obtain a broad but informative overview of protein biology. Grouping proteins into biological categories using a GO slim approach comes with the caveat that its is possible for a single protein to being represented in more than one category due to the multi-functional nature of certain proteins. Similarly some proteins that have yet to be annotated or protein whose annotations are yet to be deposited in a database may not feature in such grouping systems until such a time when the annotation is created and incorporated in the database. The same can be said of drug information. Some drugs have been known to have protein targets from more than one biological grouping due to the drug having multiple targets and/or different mechanisms of action. However with the biological information captured using GO to protein targets in ChEMBL, the GO slim still provides a quick and useful way of navigating protein target space and related small molecule information.

## Conclusion

We have created a protein target navigational tool using the ChEMBL protein target slim specifically designed for browsing drug discovery protein targets. This tool provides a rapid way of searching for biological information to proteins in a large database. The tool also allows for a rapid overview of the biology of protein target space. Besides providing information on the biological process and molecular functions of protein targets the navigation tree also readily provides an overview of protein target subcellular localisation.

The slim is freely available for use and is updated regularly to reflect changes in both the GO and ChEMBL protein target space. We anticipate the slim will be a useful tool for other researchers and tool developers wishing to display, explore and summarize GO data in the area of drug discovery.

## Availability and requirements

The ChEMBL drug target slim is freely available from the ChEMBL website https://www.ebi.ac.uk/chembl/target/browser [15]. The GO terms slim terms used for the slim classes are available from the Gene Ontology Consortium together with the other GO slims [16]. The ChEMBL data is made available on a Creative Commons Attribution-Share Alike 3.0 Unported License.

## Abbreviations

GAF: Gene association file
GO: Gene ontology
WHO ATC: World Health Organisation Anatomical Therapeutic Chemical Classification
